# His-FLAG Tag as a Fusion Partner of Glycosylated Human Interferon-Gamma and Its Mutant: Gain or Loss?

**DOI:** 10.1155/2017/3018608

**Published:** 2017-06-08

**Authors:** Elena Krachmarova, Milena Tileva, Elena Lilkova, Peicho Petkov, Klaus Maskos, Nevena Ilieva, Ivan Ivanov, Leandar Litov, Genoveva Nacheva

**Affiliations:** ^1^Institute of Molecular Biology “Roumen Tsanev”, Bulgarian Academy of Sciences, 1113 Sofia, Bulgaria; ^2^Institute for Interdisciplinary Research and Technologies, 1421 Sofia, Bulgaria; ^3^Institute of Information and Communication Technologies, Bulgarian Academy of Sciences, 1113 Sofia, Bulgaria; ^4^Faculty of Physics, Sofia University “St. Kliment Ohridski”, 1164 Sofia, Bulgaria; ^5^Proteros Biostructures, 82152 Martinsried, Germany

## Abstract

In order to obtain glycosylated human interferon-gamma (hIFN*γ*) and its highly prone to aggregation mutant K88Q, a secretory expression in insect cells was employed. To facilitate recombinant proteins purification, detection, and stability the baculovirus expression vectors were constructed to bear N-terminal His_6_-FLAG tag. Although the obtained proteins were glycosylated, we found that their biological activity was 100 times lower than expected. Our attempts to recover the biological properties of both proteins by tag removal failed due to enterokinase resistance of the tag. Surprisingly, the tag was easily cleaved when the proteins were expressed in* E. coli* cells and the tag-free proteins showed fully restored activity. To shed light on this phenomenon we performed molecular dynamics simulations. The latter showed that the tags interact with the receptor binding domains and the flexible C-termini of the fusion proteins thus suppressing their complex formation with the hIFN*γ* receptor. We hypothesize that in the case of glycosylated proteins the tag/C-terminal interaction positions the FLAG peptide in close proximity to the glycans thus sterically impeding the enterokinase access to its recognition site.

## 1. Introduction 

The choice of optimal expression conditions and protocol for purification of recombinant proteins depends on the characteristics of the protein of interest, the experimental needs, and the further application of the purified product. Many proteins need to be modified following translation in order to become active. A large number of recombinant proteins with clinical relevance are naturally glycosylated [1]. This posttranslational modification plays important role in protein folding, stability, and protease resistance in the blood stream and is exclusively carried out by eukaryotic cells. Many eukaryotic hosts such as mammalian, insect, yeast, and plant cells have been developed to express glycosylated proteins, and each has their relative advantages and disadvantages [2]. Although the early steps of glycosylation in the endoplasmic reticulum (ER) are very similar across eukaryotes, the structure of the final glycans is species specific due to differences in the oligosaccharide processing pathways [2, 3]. Expression of glycoproteins in mammalian cells will result in mammalian-type glycosylation. For human proteins this is ideal; however some cell lines will add the nonhuman Gal *α*1-3 Gal epitope and the *N*-glycolylneuraminic acid. Insect expression systems will add shorter *N*-glycans, with little sialylation. Plant cells typically produce glycans that contain extra fucose and xylose residues [4]. Yeast expression systems have a very different glycosylation pattern from mammalian cells, with only mannose-containing glycans [5].

Human interferon-gamma (hIFN*γ*) is a secretory glycoprotein, which plays key role in the regulation of innate and adaptive immunity. It is composed of 143 amino acid residues (17 kDa) and is a rare case of cysteinless protein. The biologically active form of hIFN*γ* is a homodimer where the two monomers are bound in antiparallel direction. The dimeric structure is stabilized by the intertwining of helices across the subunit interface with multiple intersubunit interactions [6]. The natural hIFN*γ* carries two N-glycosylation sites (Asn25 and Asn97), which are exposed to the surface of the homodimer. Two forms of this cytokine with apparent molecular weight of 20 and 25 kDa were isolated from peripheral blood lymphocytes. The 25 kDa fraction contained carbohydrates on both of its glycosylation sites, while the 20 kDa fraction was monoglycosylated [7]. Glycosylation does not affect the biological activity but is essential for the solubility and protease resistance of hIFN*γ*. The glycan residues, especially those at Asn25, play an important role in protease resistance to granulocyte proteases, purified elastase, cathepsin G, and plasmin [8].

During the recent years we have been developing mutant analogues of the hIFN*γ* to serve as its inactive antagonists in the treatment of autoimmune diseases, whose aetiology is related to the abnormal expression of this cytokine [9, 10]. Among the great variety of constructs the mutant K88Q (Q substitution for K in position 88) was selected for further investigations [11–13]. Our experience showed that upon heterologous expression in* E. coli*, the wild-type hIFN*γ* predominantly (60%) aggregates in the form of inclusion bodies [14]. The mutant K88Q showed lower stability and higher propensity for aggregation compared to the wild-type protein under the same expression conditions. Taking into consideration that nonglycosylated proteins are less stable and more susceptible to protease attacks compared to the glycosylated ones, we aimed to produce both the wild-type hIFN*γ* and its mutant analogue as glycosylated proteins. Our previous attempts to express both proteins in mammalian cell lines HEK293 and CAP-T led to unsatisfactory protein yield and the cell cultures demonstrated poor growth and viability [15].

Insect cells have emerged in the last years as attractive choices for the expression of recombinant proteins [16]. Compared to the expression in mammalian cells, insects are preferred choice due to the fact that they have higher expression levels and still employ similar glycosylation mechanisms as the mammalian cells [17]. The most popular for production of protein therapeutics is the baculovirus expression system [18]. It was first employed in 1983 for expression of human IFN*β* [19] and since then it has been successfully used for expression of thousands of eukaryotic recombinant proteins [20].

In the resent years the coexpression of the target protein as part of a fusion has become a common approach for production of recombinant proteins in soluble and active form. The fusion partner can provide increased expression levels, greater solubility, and convenient purification process [21]. The most frequently used tags include glutathione S-transferase [22], FLAG tag [23], His-tag [24], Arg-tag [25], and maltose-binding protein [26]. The choice of tag depends on the molecular characteristics of the target protein itself, the specificity of the expression system, and the further application of the final product. Depending on the nature of the tag it can be either enzymatically [27] or chemically [28] removed from the fusion in order to obtain the target protein.

The FLAG tag is a short 8-amino-acid peptide (DYKDDDDK) that is commonly used to improve and facilitate the detection and purification of the target protein. As a hydrophilic peptide it locates on the surface of the fusion protein and therefore it is accessible to antibodies. Due to its small size and hydrophilic nature, it usually does not affect the folding and biological activity of the target protein [21]. In addition, the FLAG tag can be completely removed by enterokinase, which is specific for the five C-terminal amino acids of its sequence [29]. Thus the enzymatic cleavage results in obtaining of recombinant proteins free of any additional downstream amino acid residues.

In order to obtain soluble and stable glycosylated proteins we chose to express the wild-type hIFN*γ* and its mutant K88Q as secretory N-terminal His_6_-FLAG fusion proteins in baculovirus-infected insect* (Trichoplusia ni)* High Five® cells. The His_6_-FLAG tag was selected because it provides handy purification protocol and convenient detection of the recombinant proteins. To our knowledge there are no other publications dedicated to the expression of recombinant hIFN*γ* in this system. In the present paper we report and discuss our findings on the negative effect of His_6_-FLAG tag on the biological activity of the purified hIFN*γ* and K88Q and its resistance to enterokinase digestion when the proteins were expressed as glycoproteins in insect cell line.

## 2. Materials and Methods

### 2.1. Construction of His_6_-FLAG-hIFN*γ* and His_6_-FLAG-K88Q Vectors for Expression in Insect Cells

Codon optimized genes encoding His_6_-FLAG-hIFN*γ* and His_6_-FLAG-K88Q in pMA transfer vectors were synthesized by Life Technologies. The genes were cloned into pFastBac™1 (Invitrogen™) via* Bam*HI and* Xho*I restriction sites. The resulting constructs carried N-terminal melittin signal sequence for secretion. The bacmid-DNA was generated in* E. coli* DH10Bac and transfected into* Spodoptera frugiperda* Sf9 cells to generate recombinant baculovirus (P1-virus) according to the standard Invitrogen protocol. Viral stocks were further propagated according to the manufacturer's protocol (Invitrogen).

### 2.2. Secretory Expression in High Five™ and Purification of His_6_-FLAG-hIFN*γ* and His_6_-FLAG-K88Q Fusion Proteins

For expression of the recombinant proteins, 5 L of High Five cell culture (2 × 10^6^ cells/mL) in Grace's medium supplemented with 10% FCS and 0.1% Pluronic was infected with 100 mL of high titerless virus stock (HTVS) and grown for 48–72 hours in a 10 L CultiBag RM, (Sartorius Stedim Biotech) on bioreactor BioWave 50 SPS (Wave Biotech). The cells were harvested by centrifugation at 900 ×g for 15 min at 4°C and the supernatant was filtered through filter paper (Macherey-Nagel Filter Papers Folded MN 615 1/4, *⌀* 320 mm). After filtration stock buffer containing 1 M HEPES, pH 7.5, 2 M imidazole, and 100 mM NiSO4 was added to the supernatant to final concentrations 10 mM HEPES, 20 mM imidazole, and 1 mM NiSO4.

The affinity chromatography was performed by HisPur™ Ni-NTA Magnetic Beads (Thermo Scientific™). The beads were prewashed with buffer containing 20 mM HEPES, pH 7.5, 375 mM NaCl, 20 mM imidazole, pH 8.0, and 5% ethylene glycol, added to the filtered supernatant and incubated for 1 h at room temperature by gentle mixing. The beads were then collected by centrifugation at 500 ×g for 10 min at 4°C, the supernatant was discarded, and the beads were manually loaded on XK 16/20 Column (GE Healthcare Life Science). The chromatography was performed on ÄKTA™ purifier (GE Healthcare Life Science) at flow rate of 0.5 ml/min. Proteins were eluted with a gradient of 40–500 mM imidazole, pH 8.0 in 20 Mm HEPES, pH 7.5 containing 375 mM NaCl and 5% ethylene glycol. Fractions were analysed by SDS-PAGE and those containing His_6_-FLAG-tagged proteins were combined and subjected to gel filtration chromatography on Superdex 75 column 16/60 (GE Healthcare Life Science). The column was equilibrated with buffer containing 20 mM HEPES, pH 7.5, 150 mM NaCl and 5% ethylene glycol. The peak fractions were pooled after SDS-PAGE analysis.

### 2.3. Construction of His_6_-FLAG-hIFN*γ* and His_6_-FLAG-K88Q Vectors for Expression in* E. coli* Cells

Codon optimized synthetic genes encoding His_6_-FLAG-hIFN*γ* and His_6_-FLAG-K88Q in pMA transfer vectors were synthesized by Life Technologies. After digestion with* Nco*I and* Xho*I the products were purified and ligated to the linearised vector pET28a (Qiagen) using Rapid DNA Ligation kit (Roche) for 15 min at 20°C. TOP10F'* E. coli* cells were transformed and the positive clones were selected by kanamycin resistance (70 *µ*g/ml) followed by Colony PCR. Qiagen Qiacube Lab Robot was used for isolation of plasmid DNA which was further verified for sequence integrity by Eurofins Genomics, Germany.

### 2.4. Expression in* E. coli *Cells and Purification of His_6_-FLAG-hIFN*γ* and His_6_-FLAG-K88Q Fusion Proteins

The recombinant plasmids pET28a/His_6_-FLAG-hIFN*γ* and pET28a/His_6_-FLAG-K88Q were transformed into* E. coli* BL21(DE3) cells. Inoculum (0.1 L) was prepared by overnight cultivation of the transformed bacteria in LB medium supplemented with 70 *µ*g/ml kanamycin at 37°C. Bacteria were grown at 37°C with agitation at 120 rpm. Gene expression was induced with IPTG (0.1 mM) at OD_600_ = 0.6–0.7 and the cells were grown for 16 h at 18°C until cell density of OD_600_ = 8.0.

The cells were collected by centrifugation at 4000 ×g for 15 min at 4°C and washed with PBS. The bacterial pellet was resuspended in 100 ml of buffer A (20 mM HEPES, pH 7.5, 40 mM imidazole, pH 8.0, 375 mM NaCl, and 5% ethylene glycol) supplemented with lysozyme (1 mg/ml), DNase (10 *µ*g/ml), and 4 “Complete, EDTA-free Protease Inhibitor Cocktail Tablets” (Roche Applied Science). After incubation on ice for 30 min the cells were disrupted on Branson Sonifier 250 (4 cycles of 1 min pulses at amplitude 40%). Cell lysates were centrifuged at 75,000 ×g for 40 min at 4°C and both fractions (supernatant and pellet) were analysed by SDS-PAGE. Supernatants were loaded on 10 ml prepacked Nickel Sepharose High Performance His_6_-Trap-Sepharose column (GE Healthcare Life Science) equilibrated with buffer A. The chromatography was carried out on an ÄKTA purifier (GE Healthcare Life Science) at flow rate of 2 ml/min. Proteins were eluted with a gradient of 40–500 mM imidazole, pH 8.0 in 20 Mm HEPES, pH 7.5, containing 375 mM NaCl and 5% ethylene glycol. Fractions were analysed by SDS-PAGE and those containing His_6_-FLAG-tagged protein were combined and subjected to gel filtration chromatography on Superdex 75 column 16/60 (GE Healthcare Life Science). The column was equilibrated with buffer containing 20 mM Hepes, pH 7.5, 150 mM NaCl, and 5% ethylene glycol. The peak fractions were pooled after SDS-PAGE analysis.

### 2.5. Proteolytic Digestion of the His_6_-FLAG Tag and Purification of the Cleaved Proteins

The purified bacterial and insect proteins were subjected to proteolytic digestion by enterokinase (Roche) in buffer containing 20 mM HEPES, pH 7.5, 150 mM NaCl, and 5% ethylene glycol at protein: protease mass ratio of 1 : 20. The cleaved His_6_-FLAG tag was removed by affinity chromatography using 10 ml prepacked Nickel Sepharose High Performance His-Trap-Sepharose column (GE Healthcare Life Science) equilibrated with buffer A (20 mM HEPES pH 7.5, 375 mM NaCl, 5% ethylene glycol, and 40 mM imidazole). The proteins were eluted with a gradient of 40–500 mM imidazole, pH 8.0 in 20 mM HEPES, pH 7.5 containing 375 mM NaCl and 5% ethylene glycol and the fractions were analysed by SDS-PAGE. The protein concentration was determined by Bradford, aliquoted, and stored at −80°C for further analysis.

### 2.6. Determination of hIFN*γ* and K88Q Biological Activity

Antiproliferative activity was determined by a modified kynurenine bioassay on WISH cells as described in [30].

### 2.7. Molecular Dynamic Simulations

#### 2.7.1. Input Structures

There are 5 available structures of hIFN*γ* in PDB. For the in silico investigations we used as input the crystallographic structural data obtained with recombinant hIFN*γ* homodimer in complex with the extracellular part of its receptor hIFNGR1 (PDB ID 1FG9) [31]. Our choice was motivated by the following considerations: this is the only structure that contains the native form of the cytokine coupled with two hIFNGR1 receptors. In addition, with its 2.9 Å resolution, it is the highest quality structure available. The third receptor presented there does not interact with hIFN*γ* and is loosely coupled with the other two receptors, thus not influencing significantly the hIFN*γ* 3D structure.

**Figure 1:**
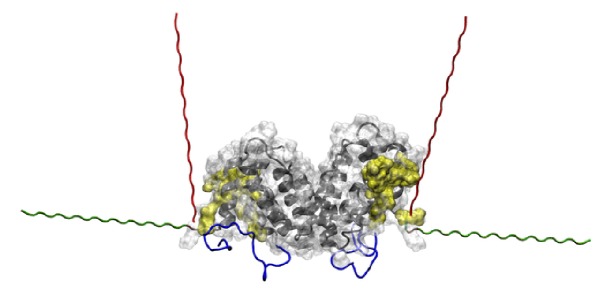
His_6_-FLAG tag initial geometries. The globule of the hIFN*γ* homodimer is depicted in grey ribbons and its molecular surface, in white bubbles. The binding interfaces for each of the two receptor subunits are shown in yellow bubbles. The previously reconstructed C-termini are in blue. The added N-terminal peptides are in green for conformation G1 and in red for conformation G2.

In the 1FG9 structure, the coordinates of the hIFN*γ* atoms are resolved up to amino acid 126, while the last 18 residues of each monomer are missing. The missing C-termini were reconstructed as described in detail in [32]. We used as a starting structure for the simulations of a His_6_-FLAG-hIFN*γ* fusion protein a model of the full length native hIFN*γ* dimer, the centroid of the largest cluster in the folding simulations in [32]. The full amino acid sequence (MGSSHHHHHHGSDYKDDDDK) of the tag was added to the N-termini of each of the fully reconstructed 143 amino acid long monomers of hIFN*γ*. The peptide was constructed in a completely extended conformation. Since there is no a priori knowledge of the conformation of this peptide, two separate simulations were performed with two different initial geometries. The two initial conformations (denoted G1 and G2) are presented in Figure 1. Two separate simulations were performed with the two initial conformations.

#### 2.7.2. MD Simulation Protocol

The simulations were done using the GROMACS MD simulation package, version 2016.1 [33–35]. The proteins were described with the CHARMM36 force filed [36], combined with the modified TIP3P water model. The systems were solvated in rectangular simulation boxes with a minimum distance between the proteins and the box of 2 nm and periodic boundary conditions were imposed. Sodium and chlorine ions with concentration 0.15 mol/l were added to neutralize the net charge of each system. Constraints [37, 38] were imposed on all bonds to allow for a 2 fs time step of the leap frog integrator. The simulations were performed in the isothermic-isobaric ensemble at a temperature of 310 K maintained by a v-rescale thermostat [39] with a coupling constant of 0.25 ps and a 1 atm pressure maintained by a Parrinello—Rahman barostat [40] with a coupling constant of 1 ps. Neighbor lists were updated every 10 steps. The PME method [41] was used for the electrostatics with a cut-off for the direct summation of 1.2 nm. Van der Waals interactions were shifted from 1.0 nm and cut at 12 Å. The configuration was written every 200 ps, amounting to 2000 frames for 100 ns simulation time.

## 3. Results

### 3.1. Secretory Expression in* High Five* Cells and Purification of His_6_-FLAG-hIFN*γ* and His_6_-FLAG-K88Q Fusion Proteins

**Figure 2:**
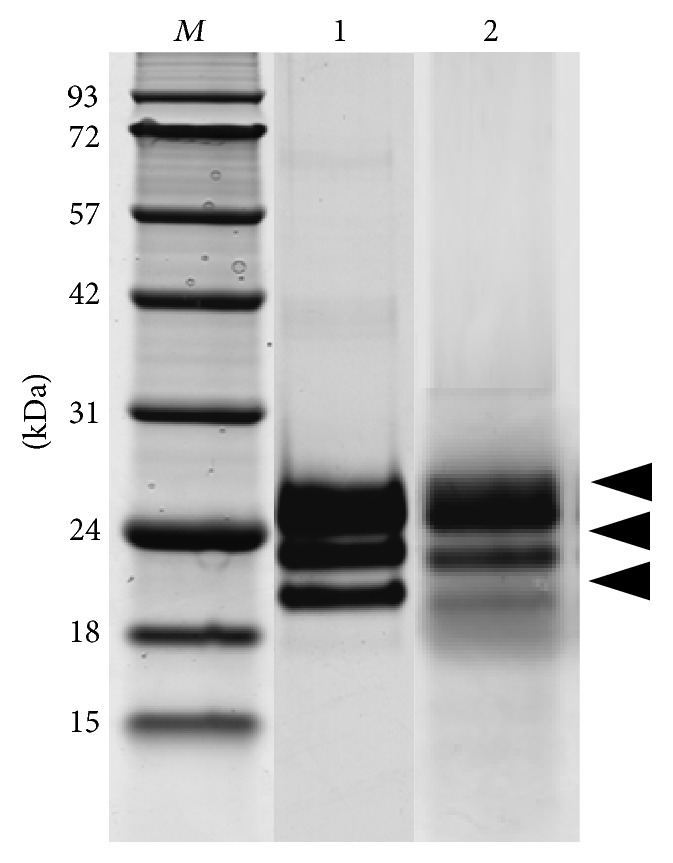
SDS-PAGE analysis of the purified target proteins expressed in High Five cells.* Lane 1: *His_6_-FLAG-hIFN*γ*;* Lane 2: *His_6_-FLAG-K88Q;* Lane M:* protein molecular weight markers in kDa. The arrows indicate the position of the glycosylated target proteins.

The expression of the two proteins hIFN*γ* and its mutant K88Q in High Five insect cells was realized by using the secretion expression vector pFastBac™1 bearing strong polyhedrin promoter. To facilitate the purification procedure His_6_ tag followed by FLAG tag was fused at the N-terminus of the target proteins. This allowed the use HisPur Ni-NTA Magnetic Beads for affinity purification of the fusion proteins secreted in the supernatant. The pooled fractions from the affinity chromatography thus obtained were concentrated and further purified by gel filtration chromatography from protein aggregates and remaining contaminants, including imidazole that might compromise the target proteins during long-term storage [42]. This two-step purification procedure led to 90% purity of both target proteins hIFN*γ* and K88Q. The yield of both secreted target proteins from the transfected High Five cells was 8–10 mg/L. They migrated in SDS-PAGE as three distinct bends related to the different extend of glycosylation (Figure 2).

### 3.2. Biological Activity of His_6_-FLAG-hIFN*γ* and His_6_-FLAG-K88Q Fusion Proteins

The biological activity of the purified glycosylated His_6_-FLAG-tagged proteins was tested by modified kynurenine bioassay [30]. The assay is based on the antiproliferative activity of hIFN*γ* and is related to the induction of indoleamine-2,3-dioxygenase (IDO), which is the first and rate-limiting enzyme in the tryptophan catabolism, catalyzing oxidative cleavage of tryptophan to N-formylkynurenine. In a following process of hydrolysis, the latter is transformed into kynurenine, which after reaction with Ehrlichs' reagent results in yellow-colored compound absorbing at 490 nm. The latter was measured in 96-well plates seeded with monolayer of WISH cells and treated with increasing concentrations of purified His_6_-FLAG-hIFN*γ* and His_6_-FLAG-K88Q proteins. The assay was run in parallel with a referent hIFN*γ* purified from bacterial inclusion bodies and having specific biological activity of 2–5 × 10^7^ IU/mg [43]. Surprisingly, both His_6_-FLAG-tagged proteins showed specific activity, which was two orders of magnitude lower than the expected one [44]. It was concluded that the His_6_-FLAG tag interferes with the biological activity of hIFN*γ* and its mutant K88Q.

### 3.3. Proteolytic Digestion of His_6_-FLAG-hIFN*γ* and His_6_-FLAG-K88Q Fusion Proteins Expressed in High Five Cells

**Figure 3:**
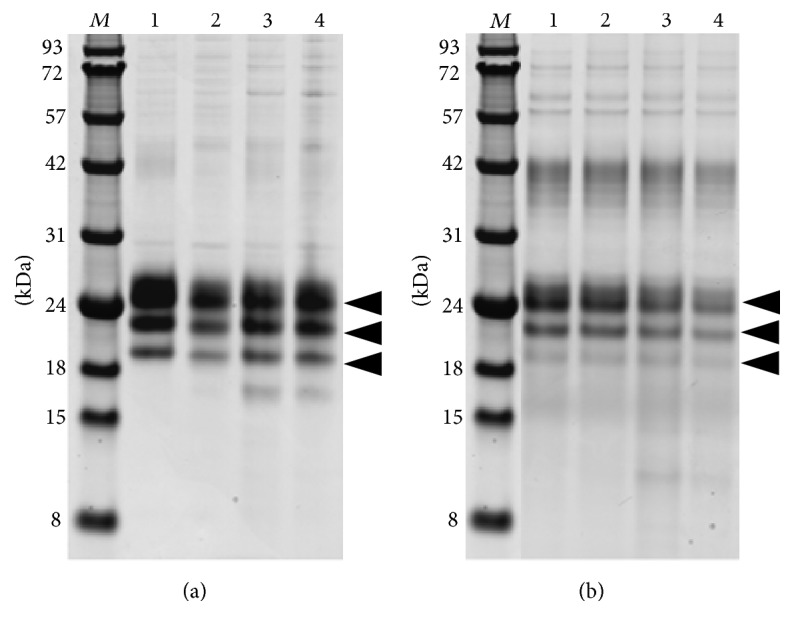
SDS-PAGE analysis of His_6_-FLAG tag cleavage of His_6_-FLAG-hIFN*γ* (a) and His-FLAG-K88Q (b) fusion proteins expressed in High Five cells. The incubation was carried out in buffer containing 20 mM HEPES, pH 7.5, 150 mM NaCl, and 5% ethylene glycol supplemented with different additives.* Lane 1*: untreated sample;* Lane 2*: 1 U recombinant enterokinase with 2 mM CaCl_2_;* Lane 3*: 1 U native enterokinase with 2 M urea;* Lane 4:* 1 U native enterokinase with 300 mM arginine.* Lane M*: protein molecular weight markers in kDa. The arrows indicate the position of the glycosylated target proteins.

In order to recover the biological activity of the two glycosylated proteins they were treated with enterokinase recognizing the oligopeptide sequence DDDDK. For our pilot digestion experiments we used two different types of enterokinase: (i) native form (Roche) and (ii) recombinant light chain of porcine enterokinase (GenScript). In order to optimize the digestion conditions we varied the reaction parameters as follows: (1) enzyme per reaction mixture: 0.1 and 1 U; (2) incubation temperature: 4°C, room temperature, and 37°C; (3) composition of the reaction buffer. In the latter case the buffer additives were chosen according to the recommendations of the manufacturers. They included 2 mM CaCl_2_; urea 1, 2, and 3 M; triton X-100, 0,01, 0,1, and 1%; and acetonitrile, 5 and 10%. Since both target proteins are aggregation prone, additional additives that are known to affect the protein stability were also tested: arginine, 0.1, 0.3, and 1 M, and potassium glutamate monohydrate, 30, 100, and 300 mM. All incubations were carried over night. Unexpectedly, despite the high number of variation in the reaction conditions, none of them resulted in cleavage of the His_6_-FLAG tag from both glycosylated proteins. Examples of some of the digestion conditions are presented in Figure 3.

### 3.4. Bacterial Expression, Purification, and Biological Activity of His_6_-FLAG-hIFN*γ* and His_6_-FLAG-K88Q Fusion Proteins

**Figure 4:**
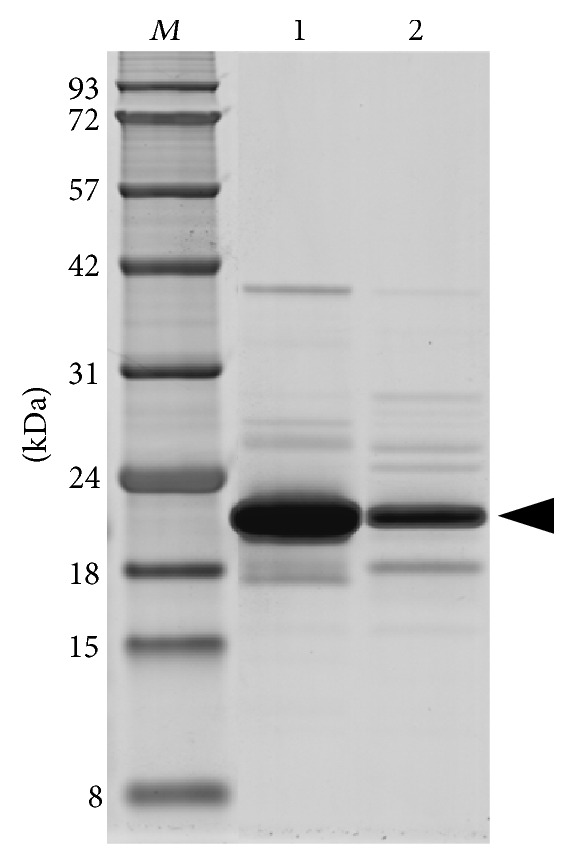
SDS-PAGE analysis of the purified target proteins expressed in* E. coli* BL21(DE3).* Lane 1:* His_6_-FLAG-hIFN*γ*;* Lane 2: *His_6_-FLAG-K88Q;* Lane M:* protein molecular weight markers in kDa. The arrow indicates the position of target recombinant proteins.

To clarify the reasons for the enterokinase resistance of His_6_-FLAG-hIFN*γ* and His_6_-FLAG-K88Q fusion proteins expressed in insect cells proteins we performed inducible expression of the same His_6_-FLAG-tagged fusion genes in* E. coli *strain BL21(DE) cells. Since the recombinant proteins bore the same N-terminal fusion partner as in the case of the insect expression, identical purification strategy was applied. The obtained proteins were with purity of 85–90% and expectedly migrated in one distinct band during the SDS-PAGE (Figure 4).

The purified from bacterial supernatants His_6_-FLAG-hIFN*γ* and His_6_-FLAG-K88Q showed 100 times lower biological activity, that is, similar to that of the fusion proteins obtained from insect cells. This result confirmed our conclusion that the His_6_-FLAG tag interferes with the biological activity of both investigated proteins independently of the expression system used.

### 3.5. MD Simulations of His_6_-FLAG-hIFN*γ* Fusion Protein

**Figure 5:**
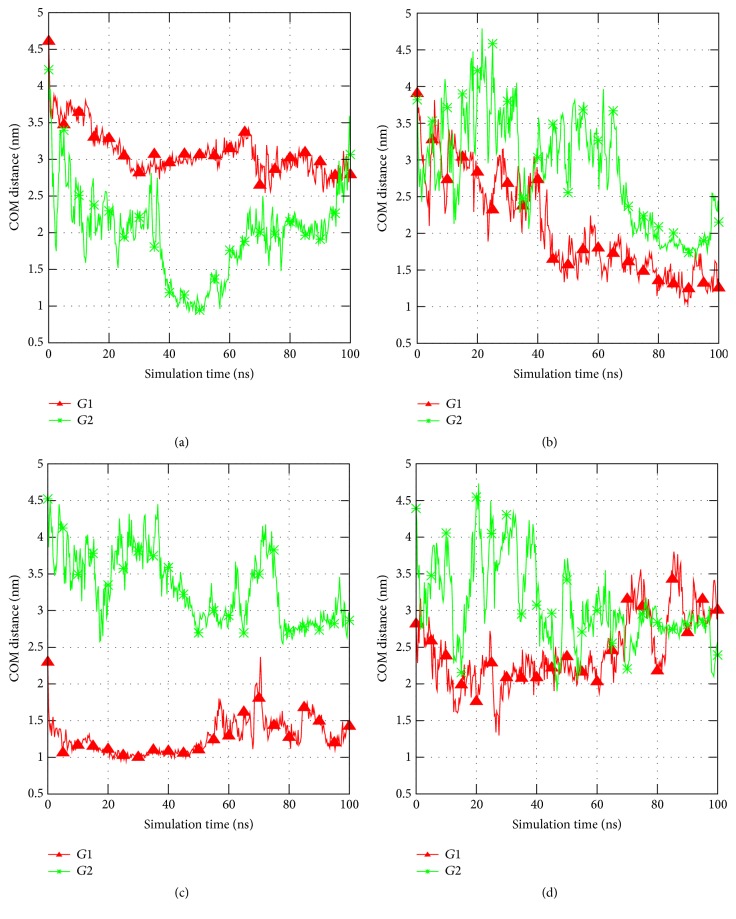
Distances between the centers-of-mass of (a) N-terminal peptide in chain A and the C-terminus of chain B; (b) N-terminal peptide in chain B and the C-terminus in chain A; (c) N-terminal peptide in chain A and the receptor binding site in AB loop of chain B; (d) N-terminal peptide in chain B and the receptor binding site in AB loop of chain A.

In order to shed light on the reasons for the His_6_-FLAG interference with the biological activity of both fusion proteins we performed MD simulation of the His_6_-FLAG-hIFN*γ*. During the time of the simulation (100 ns) no alterations in the structure of hIFN*γ* receptor binding interfaces (residues 18–26 in the AB loop, residues 109–111 and 115–118 [31]) were observed when the two N-terminal His_6_-FLAG tag peptides were added. Instead, the peptides began to fold and to interact with the globule and with the C-termini of the cytokine. This is demonstrated in the plots of the distances between the centers-of-mass of the N-terminal peptides and both C-termini, as well as the N-terminal peptides and the receptor binding interfaces, presented in Figure 5. Due to the electrostatic attraction the N-tags start to approach hIFN*γ* C-terminal tails (Figures 5(a) and 5(b)). The interaction with the globule is not that intensive, but the peptides remain fairly close to the receptor binding sites (Figures 5(c) and 5(d)).

**Figure 6:**
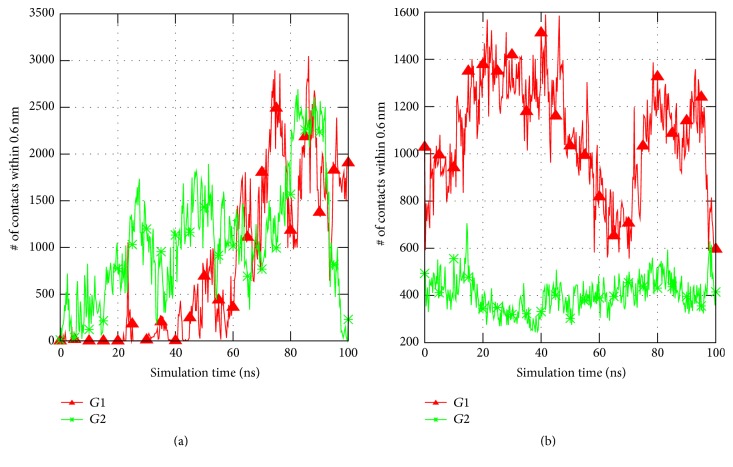
Number of native contacts between the atoms of (a) N-terminal peptides and the C-termini of hIFN*γ*; (b) N-terminal peptides and the receptor binding interfaces. A contact was considered present, if the distance between the pair of atoms is less than 0.6 nm.

In both G1 and G2 simulations the tag peptides form multiple contacts with the cytokine. The number of native contacts between the N-tags and the C-termini or the binding sites is shown in Figure 6. On average, about 400 contacts are formed between the binding sites and the N-tags in the simulation, initiated from the G2 conformation. In the simulation starting from the G1 configuration a significantly higher number of contacts are present but they drop notably by the end of the simulation, about the same 400. This is probably due to the starting configuration, since in the G1 geometry the N-terminal peptides are much closer to the binding sites than in the G2 configuration.

**Figure 7:**
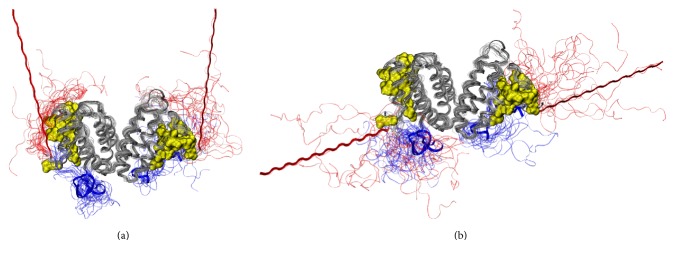
Summary of the trajectories. The conformations of the N-terminal peptides are shown in red and of the C-termini, in blue. The globule of hIFN*γ* is in grey ribbon and the receptor binding sites, in yellow bubbles. In the simulations starting from *G*1 (a), the added peptides remain predominantly close to the globule and the binding sites in particular. In the *G*2 simulation (b), one of the N-terminal peptides remains fairly distant to the globule and interacts loosely with the respective C-terminus, whereas the peptide in the other monomer approaches the globule.

A presentation of the conformations, adopted by the His_6_-FLAG tags around the hIFN*γ* homodimer is given in Figure 7.

Since the introduced mutation in K88Q lies away from the receptor binding sites and the structure of K88Q is not affected by the mutation we believe that the data from the simulations performed with the wild-type hIFN*γ* are valid for the mutant K88Q.

### 3.6. Proteolytic Digestion of the His_6_-FLAG and Biological Activity of hIFN*γ* and K88Q Proteins after the Tag Removal

**Figure 8:**
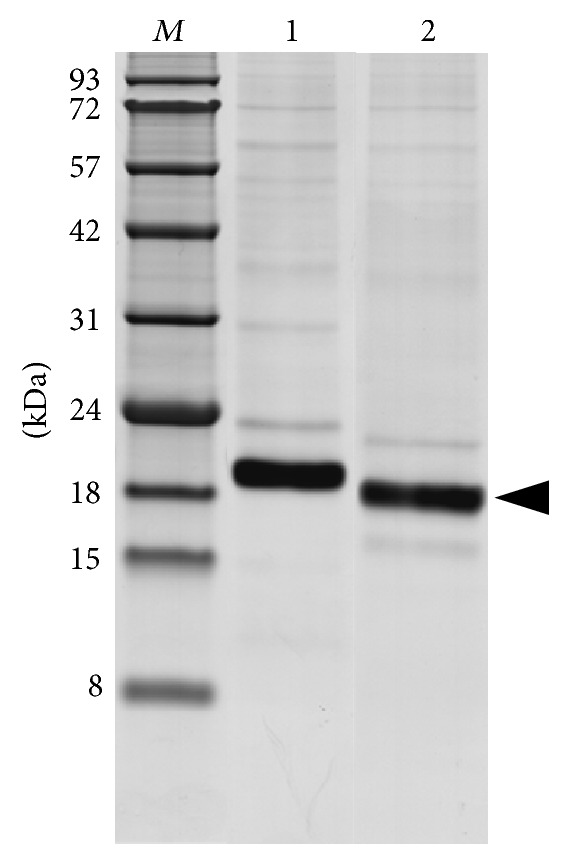
SDS-PAGE of hIFN*γ* after His_6_-FLAG tag cleavage and IMAC chromatography.* Lane 1:* fraction before digestion;* Lane 2: *eluted fraction containing cleaved hIFN*γ*;* Lane M:* protein molecular weight markers in kDa. The arrow indicates the position of the cleaved hIFN*γ*.

By performing the previously described pilot digestion experiments, optimal cleavage was observed by using native enterokinase at protein: protease mass ratio of 1 : 20 after overnight incubation at room temperature. The cleaved His_6_-FLAG tag as well as the uncleaved proteins were removed completely from the untagged proteins by affinity chromatography where the cleaved hIFN*γ* (Figure 8) and K88Q (data not shown) were obtained in the flow through fractions.

The results from the kynurenine bioassay showed that the biological activity of hIFN*γ* and K88Q expressed in* E. coli* cells was fully restored after the tag removal. The activity of hIFN*γ* was comparable to that of the referent sample (3 × 10^7^ IU/mg), and the activity of the mutant K88Q was 2.5 × 10^5^ IU/mg, which is in agreement with the value we have obtained by using SUMO fusion technology assisted by coexpression of chaperones [44].

## 4. Discussion

N-glycosylation affects positively the solubility of hIFN*γ*, its protease resistance, and circulation half-life in the bloodstream [45]. Taking into consideration the unsatisfactory results from our previous attempts to express glycosylated hIFN*γ* in human cell lines (HEK293 and CAP-T) [15] in this study we employed baculovirus-infected insect* Trichoplusia ni* BTI-Tn-5B1-4 cell line, also known as High Five, for expression of hIFN*γ* and its mutant K88Q. Our choice was based on data pointing that this cell line is more efficient for expression of recombinant proteins than other lepidopteran cell lines, such as* Spodoptera frugiperda* Sf9 cell line [46]. It is also shown that High Five cells conduct protein glycosylation in more “humanized” manner [47], which is of a great importance for production of recombinant proteins with potential application as therapeutics [1]. The employed expression vector codes for N-terminal His_6_-FLAG tag. The latter was chosen because it is believed that the original conformation of the target protein in the fusion remains unaffected and also because of the stabilizing effect of the FLAG peptide [24]. Surprisingly, our data showed that the His_6_-FLAG tag strongly interfered with the biological activity of both hIFN*γ* and K88Q expressed in both insect and bacterial cells. The measured antiproliferative activity was two orders of magnitude lower than that of the same proteins produced by the SUMO fusion technology [44]. These results are in contrast to the generally held idea that the N-terminal His_6_-FLAG tag has no effect or has negligible influence on the biological activity of the target proteins [23]. Indeed, many proteins such as transcription and growth factors, enzymes, and membrane proteins were successfully produced by the FLAG tag technology [48–51].

To explain the interference of His_6_-FLAG tag with hIFN*γ* and K88Q biological activity we performed MD simulations of the His_6_-FLAG-hIFN*γ* fusion protein structure. The results show that the addition of the two N-terminal peptides does not alter the conformation of the binding sites of the cytokine. In fact, the whole globular part of the hIFN*γ* homodimer remains very stable during both simulations. This is to be expected, since the removal of the tags fully restores the biological activity, that is, the high binding affinity of hIFN*γ* to hIFNGR1.

The receptor binding sites in hIFN*γ* to the extracellular part IFNGR1 are located in three distinct areas: (i) the loop between helices A and B (residues 18–26), (ii) His111, and (iii) a short putative area (residues 128–131) in the flexible C-terminal domain. This means that not only the N-terminus, but also the C-terminus accounts for binding of hIFN*γ* to IFNGR1 [6, 31, 52]. Our simulations suggest that the addition of the N-terminal peptides may influence the cytokine-receptor binding in two different ways. Firstly, since the His_6_-FLAG tags contain highly negatively charged cluster of four aspartic acid residues, they are attracted electrostatically by the two positively charged domains D1 (residues 125–131) and D2 (residues 137-140) in the C-termini of the cytokine. This interaction most probably contributes to the reduced affinity of hIFN*γ*, because it effectively decreases the net charge of the whole molecule and of the C-termini in particular, leading to a partial neutralization of the C-terminal domain of the cytokine. These data are in accordance with our previous observation that hIFN*γ* with completely truncated C-terminus (lacking 21 C-terminal amino acids) manifests 10-times lower biological activity [14]. It was also shown by other authors that the positively charged domains D2 and especially D1 on the C-terminus of hIFN*γ* contribute significantly to the high affinity interaction of hIFN*γ* and its receptor [53, 54]. The interaction between the N-terminal His_6_-FLAG tags and the C-termini in our simulations is rather intense. It should be noted, however, that longer simulations may demonstrate even better the significance of the electrostatic interaction between the added negatively charged N-terminal peptides and the highly positively charged C-termini of hIFN*γ* for the observed reduction in its binding affinity.

As expected, in the simulation starting from the G1 geometry, the peptides interact with the globular part of hIFN*γ* and in particular with the receptor binding interfaces. In this initial geometry they lie very close to each other. Surprisingly, even when the simulation starts from the second conformation G2, the N-terminal His_6_-FLAG tags form a fairly large amount of contacts with the receptor binding interfaces in the globule and also with the flexible positively charged C-termini. The interaction of the N-terminal peptides and the receptor binding sites in the globule of hIFN*γ* is another way for the His_6_-FLAG tag to cause a decrease in the binding affinity. This interaction is not intense but somewhat loose. The His_6_-FLAG tags do not bind the globule but remain at a distance of a few Angstroms. However, this is sufficient for them to “shield” the binding sites of hIFN*γ* and to hinder sterically the proper contact between hIFN*γ* and its receptor.

An inhibitory effect of the FLAG tag on the target protein activity or function was also reported by other authors. Papakonstantinou and coauthors showed that the introduction of FLAG tag in human activin A led to a decrease in biological activity, which was restored upon removal of the tag [55]. They assumed that the acidic nature of the FLAG tag may compromise the ability of the recombinant activin A to efficiently interact with a protein within the membrane receptor complex that acts as a transducer of activin signals [56]. The same negative effect on the protein properties was observed with a FLAG-tagged coat protein of the bacteriophage M2, which was unable to assemble into virus-like particle [57]. The main reason was attributed to the high density of negatively charged aspartate residues in the FLAG peptide.

In order to restore the biological activity of hIFN*γ* and K88Q we performed enzymatic digestion of the bacterial and insect fusion proteins. Surprisingly, we found that the fusion proteins obtained from insect cells were resistant to enterokinase independently of the enzyme source and experimental conditions, whereas the proteins isolated from* E. coli* were susceptible; the tag was successfully removed and the biological activity was fully restored. Hosfield and Lu have studied the cleavage efficiency of the enterokinase depending on the amino acids following its recognition site. They showed that only proline and tryptophan were not well tolerated when they were located downstream of the recognition sequence [58]. Since the amino acid in position P1′ in the FLAG-tagged hIFN*γ* and K88Q is glutamine the ineffective cleavage cannot be explained by unfavorable amino acids context.

**Figure 9:**
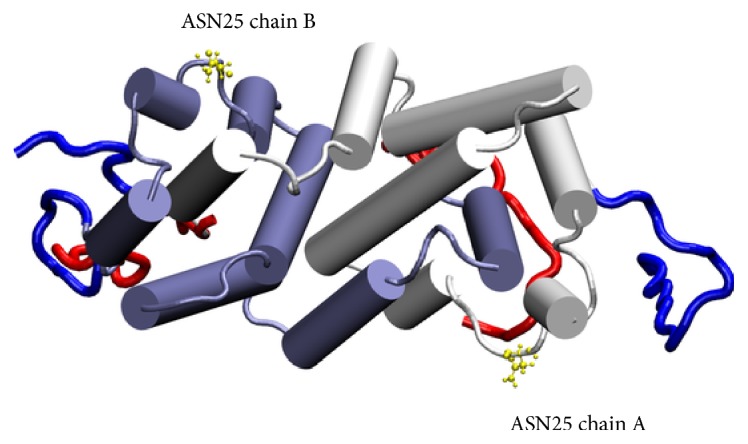
Selected frame of the trajectories of the N-terminal His_6_-FLAG tags (red) and of the C-termini (blue). The positions of Asn25 are presented in yellow. The scheme is based on the crystallographic structure of the complex hIFN*γ*/IFNGR1 where additional 3_10_ helix is formed in the AB loop.

One possible reason for the ineffective enzyme cleavage lies in the potential posttranslational modifications that the amino acids in the His_6_-FLAG tag can undergo when expressed in eukaryotic cells. Schmidt and coauthors described tyrosine sulfation of the FLAG tag that interfered with the FLAG-anti-FLAG antibodies interaction [59]. However, we are more inclined to explain the enterokinase resistance of the fusion protein from insect cells with the specific posttranslational glycosylation of hIFN*γ* proteins. There are direct data showing that the accessibility of the FLAG tag correlates with the extent of the protein glycosylation. Müller and coauthors showed that the removal of the glycosylation sites in the molecule of human multidrug resistance protein (MRP1) led to better recognition of the FLAG epitope (DYK) by the anti-FLAG monoclonal antibody [60]. Sareneva et al. [8] have shown that the glycans associated with Asn25 are essential for the protease resistance of hIFN*γ* expressed both in leukocyte culture and in Sf 9 insect cells. In contrast, the nonglycosylated hIFN*γ* expressed in* E. coli* and the mutant (N25Q, N97Q) expressed in Sf 9 cells were highly susceptible to all tested serine proteases: granulocyte protease, cathepsin G, elastase, and plasmin. Since each of the complex glycan residues increase the molecular mass of hIFN*γ* by approximately 3-4 kDa, the authors proposed that the carbohydrates most probably cover relatively large area on the dimer surface thus causing inaccessibility of the corresponding regions to the proteases. Thus the glycan residues at Asn25 may sterically impede the protease access into the vicinity of helixes A and B of one monomer and F′ of the other. These findings of Sareneva's group are even more valid for the system reported here, as Sf 9 insect cells produce oligomannose type glycans, whereas High Five are known to process much larger and complex glycans [47]. In addition, unlike the tested serine proteases, the enterokinase recognizes pentapeptide (DDDDY) rather than a single amino acid residue, meaning that masking of even one of the amino acids in the target site could cause resistance against enterokinase. Our MD simulations indicate that most probably the His_6_-FLAG tag interacts with the C-termini of hIFN*γ* and K88Q that positions it in the vicinity of a-helixes A and F′ (A′ and F, resp.) (Figure 9). Moreover, it was calculated that the distance between the four aspartic acids in the recognition site of the enterokinase and Asn25 slightly varies in the borders of 3 nm. Therefore it is logical to assume that the His_6_-FLAG tag itself is fully or partly shielded by the glycans associated with Asn25 residue.

## 5. Conclusions

In this paper we describe the negative effect of the N-terminal His_6_-FLAG tag on the biological activity of two proteins with therapeutic application (hIFN*γ* and its mutant K88Q) and its resistance to enterokinase digestion when the proteins are glycosylated. To our knowledge, we apply for the first time the His_6_-FLAG tag technology for expression of hIFN*γ* in insect cells (High Five) and explain the inhibitory effect of the tag by MD simulations of the fusion protein structure.

Although the fusion tag technology became very popular during the last decade and was successfully employed for expression of a great number of recombinant proteins, the reported here results indicate that it is not generally applicable. There is risk of failure which is due to the individual molecular structure and properties of the targeted protein.
